# Migraine-Type Headache and Functional Gastrointestinal Symptoms of Unconfirmed Aetiology in a Paediatric Patient Managed with Knee Chest Upper Cervical Chiropractic Care: A Case Report

**DOI:** 10.7759/cureus.117285

**Published:** 2026-10-01

**Authors:** Shaan Rai

**Affiliations:** 1 Chiropractic Medicine, Vitality Chiropractic Centres, Singapore, SGP

**Keywords:** anterior head carriage, central abdominal pain, cervicogenic headaches, knee chest upper cervical technique, migraine, morning headache, paediatric gastrointestinal, paediatric migraine, upper cervical chiropractic, vitality chiropractic singapore

## Abstract

An eight-year-old female child was brought to our upper cervical chiropractic clinic in Singapore by her mother with a several-month history of central abdominal pain, morning headaches with temporal pain, neck pain radiating to the arm with numbness, and episodic vomiting. She had previously been assessed by a general practitioner, who described the headache as a differential of tension-type headache versus possible migraine, not a confirmed diagnosis, and by a gastroenterologist, who arrived at a working diagnosis of gastritis based on symptoms and stool analysis alone, without endoscopic or biopsy confirmation; no advanced imaging was pursued, as the attending physician did not consider it necessary. Examination revealed anterior head carriage, a right head tilt, restricted left lateral flexion and right rotation, a short right leg on prone testing that normalised with cervical rotation, and weakness on manual muscle testing with cervical rotation: findings consistent with, though not diagnostic of, upper cervical joint dysfunction. Radiographs showed loss of normal cervical lordosis without bony or discal abnormality. The patient received knee chest upper cervical chiropractic (KCUC) care, beginning with an instrument-assisted technique and transitioning to gentle manual correction as tolerated, over approximately 16 months before relocating overseas. The family reported improvement in arm symptoms, posture, and abdominal pain, with recurrence of abdominal symptoms coinciding with a period of reduced visit adherence. As no neuroimaging was performed and no validated outcome measure was used, this temporally associated improvement cannot be distinguished from the natural history of an undiagnosed condition. This case is presented as a hypothesis-generating account of a paediatric patient with an unconfirmed migraine-type headache and functional gastrointestinal symptoms managed alongside conventional medical care, and does not establish a causal or therapeutic claim.

## Introduction

Migraine and recurrent headache are increasingly recognised in the paediatric population, and are frequently accompanied by gastrointestinal symptoms such as recurrent abdominal pain, nausea, and vomiting, which are described within the spectrum of episodic syndromes associated with migraine [1]. Paediatric presentations of this kind can be diagnostically and therapeutically challenging, often involving multiple specialists and a period of symptomatic medication before an underlying pattern is recognised.

Knee chest upper cervical chiropractic (KCUC) is an analysis and technique-specific form of chiropractic care directed at the atlanto-occipital and atlanto-axial region. Correction is delivered with the patient prone in a knee-chest position with the head rotated to one side, using a precise, low-force, high-velocity low-amplitude thrust; listings are initially assessed through postural analysis, leg-length assessment, manual muscle testing, balance testing, and thermography (bilateral paraspinal skin temperature differential), with radiographic confirmation before any correction is delivered. In paediatric patients, instrument-assisted (activator) technique may be used in place of, or preceding, manual correction.

Upper cervical and cervical manual therapies have been studied across several headache subtypes. Systematic review evidence suggests manual therapies, including chiropractic spinal manipulation, may be comparable to pharmacological prophylaxis for migraine [2], while a multi-centre randomised trial found upper cervical and upper thoracic manipulation more effective than mobilisation and exercise for cervicogenic headache in adults [3], and an observational pilot study reported improved migraine-specific quality of life following atlas realignment [4]. In children specifically, a randomised, sham-controlled trial found that chiropractic spinal manipulation reduced the frequency of recurrent headaches [5], and a case report has described resolution of chronic cervicogenic headache in a young child following spinal manipulative care [6].

Case reports describing upper cervical chiropractic care in children presenting with concurrent migraine-type headache and gastrointestinal symptoms are limited in the literature; the most directly comparable published account describes cessation of cyclic vomiting alongside headache in a seven-year-old girl following upper cervical care [7]. We report the clinical course of an eight-year-old patient presenting with this combination of symptoms, describe the changes observed during a period of chiropractic care, and discuss this within the context of the existing literature and the limitations inherent to a single paediatric case. This case adds a narrow but specific data point to a sparsely documented paediatric symptom combination and generates the hypothesis, not evidence, that upper cervical joint dysfunction may warrant consideration as a co-occurring factor in similar paediatric presentations: a hypothesis that would require a prospective, controlled study using validated paediatric outcome measures to test. This report is prepared in accordance with the CARE guidelines for case reports [8].

## Case presentation

An eight-year-old female child presented to our clinic with a symptom cluster of morning headache, episodic vomiting, central abdominal pain, and neck pain radiating into the arm with numbness. She was brought to our clinic by her mother, who reported that digestive symptoms had already been present for some time. Central abdominal pain had initially prompted assessment by a gastroenterologist, who prescribed medication and a stool softener after difficulty passing stool; stool analysis was normal. Pain medication was also prescribed for the abdominal symptoms. Around the same period, the child began experiencing temporal headache and was assessed by a general practitioner, who described the presentation as tension-type headache versus possible migraine, a differential, not a confirmed diagnosis. Neck pain developed with radiation of pain and numbness into the arm. A single episode of vomiting at school occurred, for which an anti-emetic was prescribed; nausea and morning crying on waking were also reported by the mother. The child was described by her mother as having recently begun exhibiting temper tantrums, which the mother felt reflected difficulty coping with her symptoms; the headaches were also affecting her schoolwork. The mother reported some falls while rollerblading, including minor knocks to the head, none of which had required medical attention.

This was the child's first experience of chiropractic care. The child was noted to be underweight for her age but had minimal food aversion. The child remained under the ongoing supervision of her general practitioner and gastroenterologist throughout the period described in this report. Current medications at presentation were paracetamol, ibuprofen, and an anti-emetic; specific doses and the anti-emetic's name were not recorded. There was no relevant surgical, family, or additional medical history reported. She remained physically active, participating in swimming, tennis, and rollerblading.

Clinical examination findings

Postural observation revealed anterior head carriage with the head tilted to the right. Cervical range of motion was pain-free but restricted in left lateral flexion and right rotation. Cervical distraction reproduced tenderness on palpation of the head and neck; cervical compression testing was negative. Romberg's test [9] was negative, and extraocular movements (H-pattern, convergence, divergence) were normal. Diffuse tenderness to palpation was noted throughout the cervical spine and shoulders.

A weight-bearing scale differential test (patient standing with one foot on each of two scales) showed a difference of 0.28 between left- and right-side readings (10.78 and 10.5, respectively), considered negligible. Prone leg-length assessment noted a short right leg that normalised with left cervical rotation; this was an observed finding rather than a quantified measurement. Upper cervical manual muscle testing showed weakening of arm strength on resisted testing when the head was rotated and flexed downward (looking down) to either the left or the right. Leg-length assessment and manual muscle testing are not validated diagnostic tests for upper cervical joint dysfunction, and their inter- and intra-examiner reliability is not well established; the reported normalisation of leg length with cervical rotation should therefore be regarded as an examination finding of uncertain clinical significance, given the known variability of this assessment method, rather than objective evidence of a specific diagnosis.

Investigations

Cervical radiographs (lateral cervical, base posterior, and anteroposterior open-mouth (APOM) views in neutral and lateral flexion) were obtained on Day 2 (Figures 1-5). The radiologist reported loss of normal cervical lordosis with straightening of the cervical spine, no loss of vertebral height or disc space narrowing, an unremarkable atlanto-axial articulation, and symmetrical lateral atlanto-dental intervals bilaterally.

**Figure 1:**
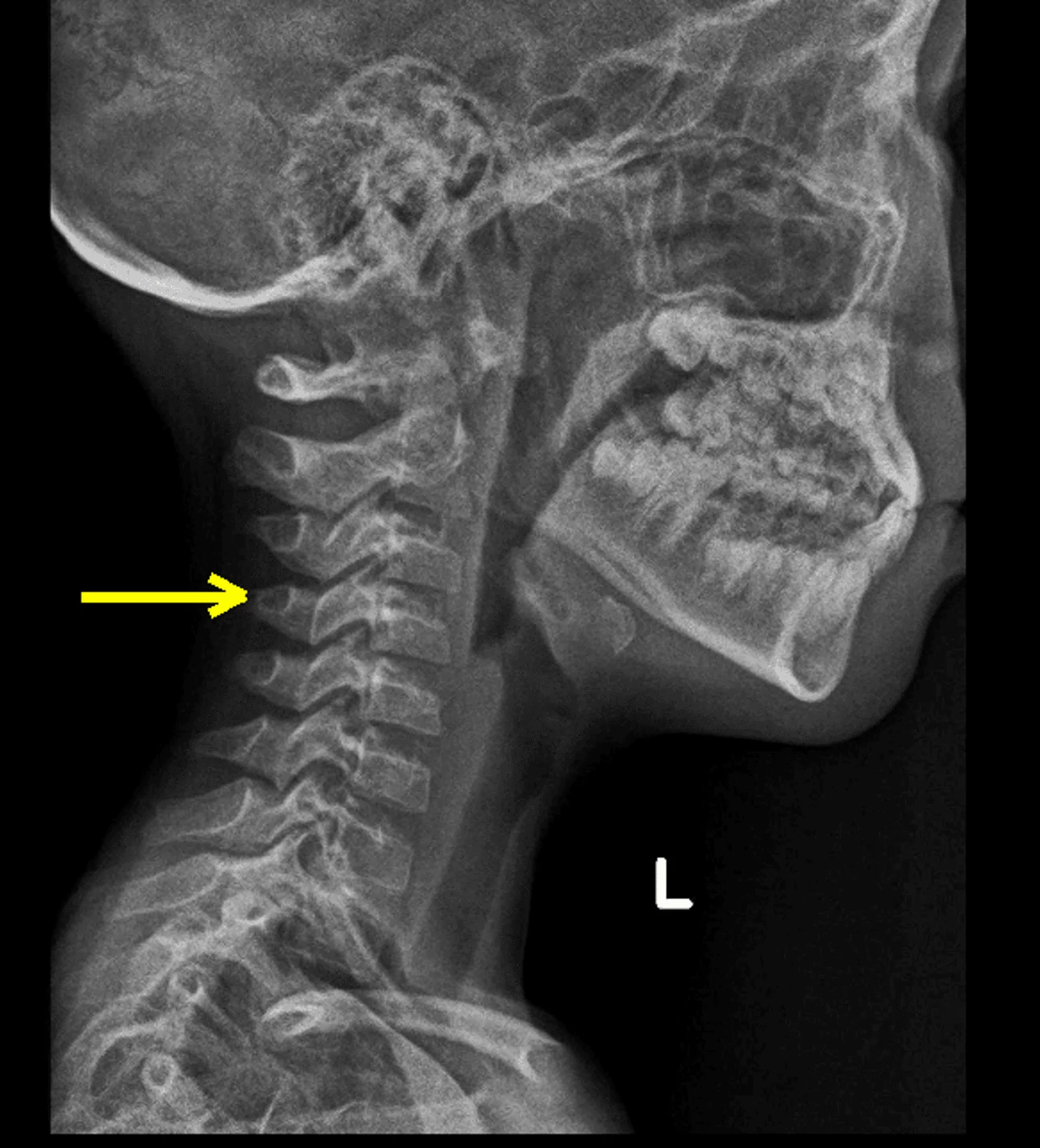
Lateral cervical radiograph showing loss of normal cervical lordosis with straightening of the cervical spine

**Figure 2:**
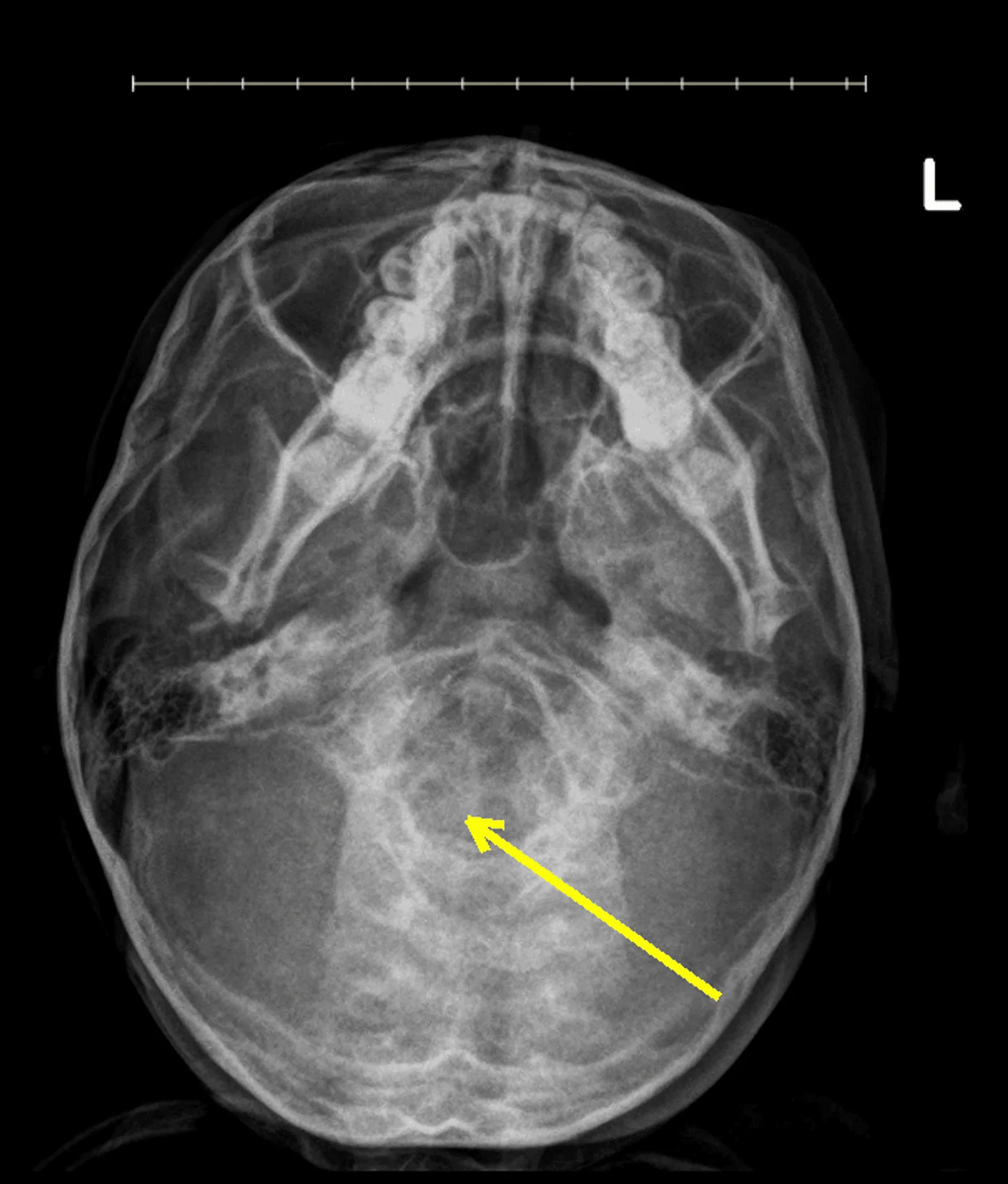
Base posterior (vertex) radiograph. Arrow indicates the upper cervical joint region.

**Figure 3:**
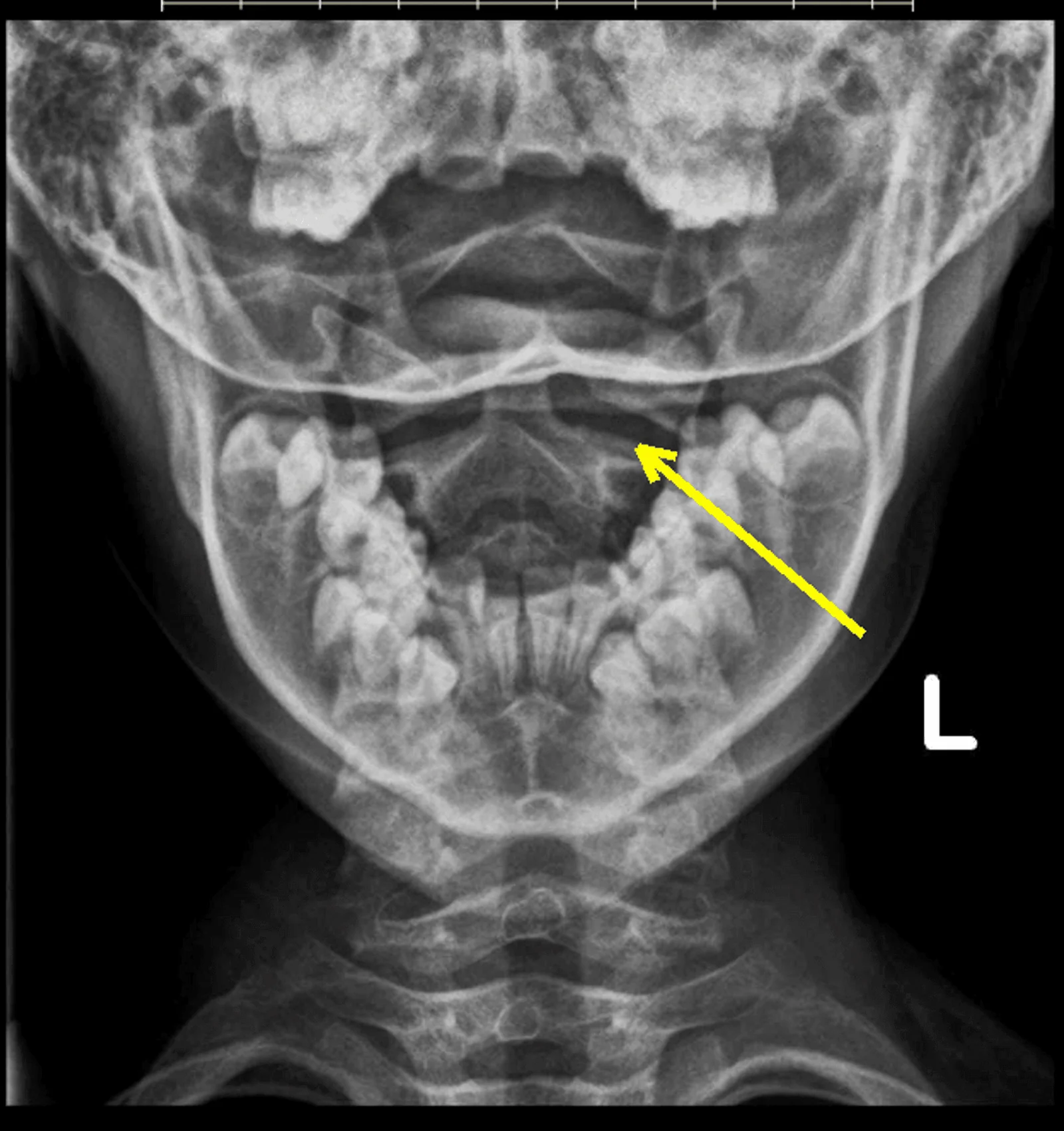
Anteroposterior open-mouth radiograph in neutral position. Arrow indicates the upper cervical region.

**Figure 4:**
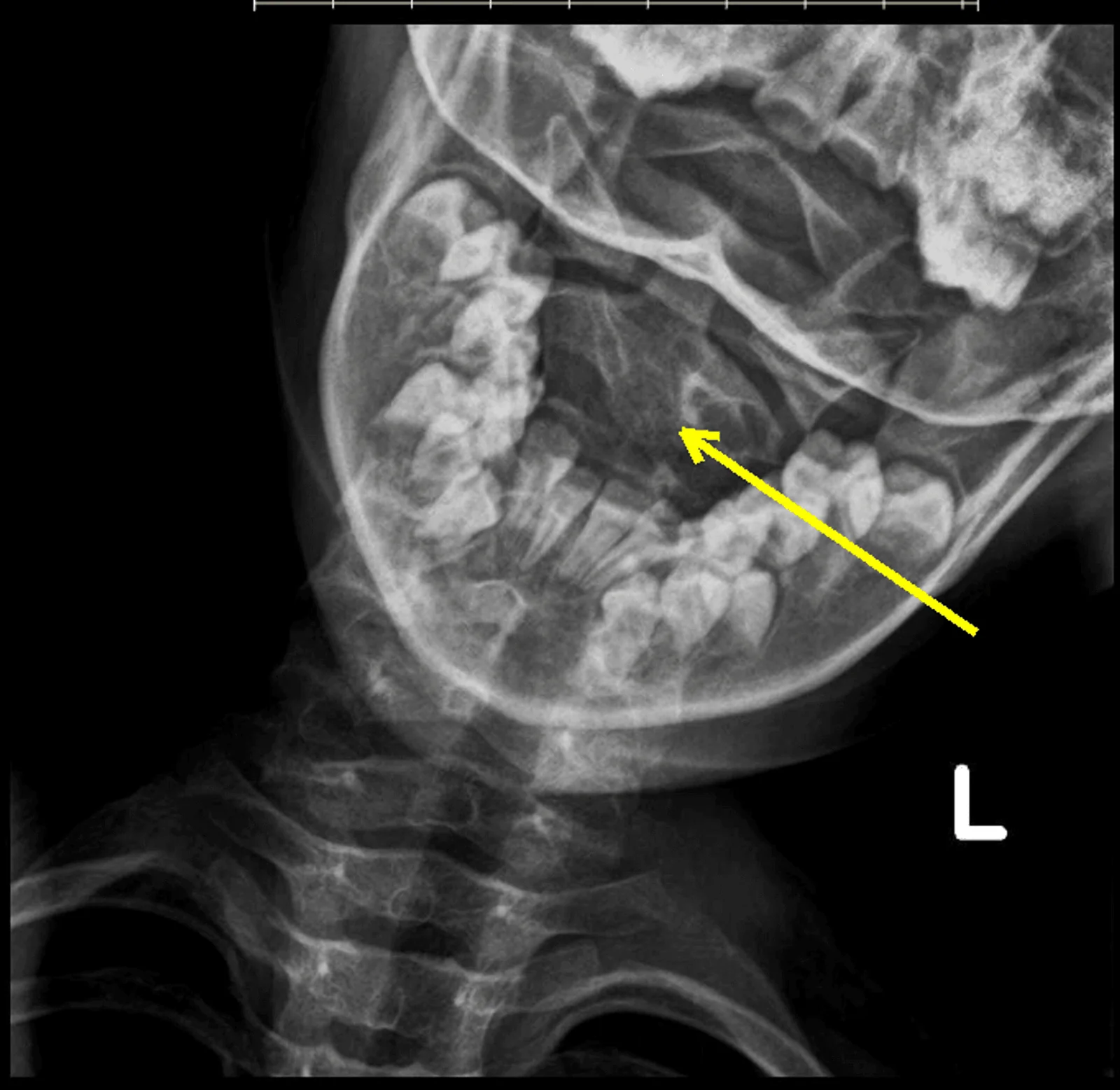
Anteroposterior open-mouth radiograph in left lateral flexion. Arrow indicates the upper cervical region.

**Figure 5:**
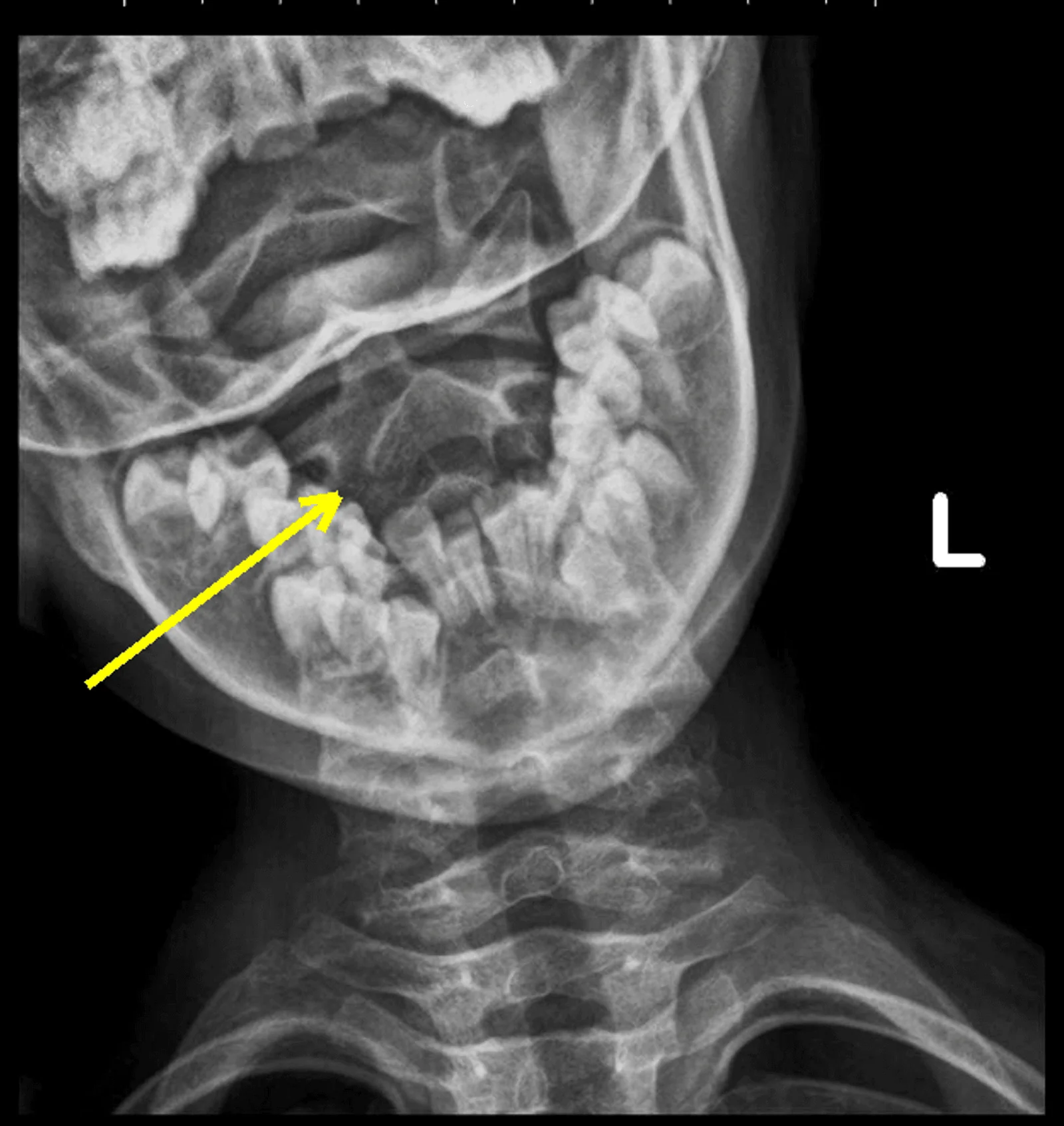
Anteroposterior open-mouth radiograph in right lateral flexion. Arrow indicates the upper cervical region.

Baseline thermography (bilateral paraspinal skin temperature differential) was performed on Day 0 and showed findings interpreted as consistent with neurophysiological irritation in the upper cervical region (Figure 6). Thermography was used in this case only as an adjunct assessment alongside the postural, orthopaedic, and radiographic findings described above, and did not independently drive treatment decisions; reliability of paraspinal thermographic assessment has been studied [10]; however, it was not a validated diagnostic or clinical outcome measure in isolation, and we cannot establish whether the thermographic changes observed over time were causally related to, or simply coincided with, the clinical changes reported elsewhere in this report. The thermographic figures are included as original clinical documentation of this case rather than as evidence of diagnostic or outcome validity.

**Figure 6:**
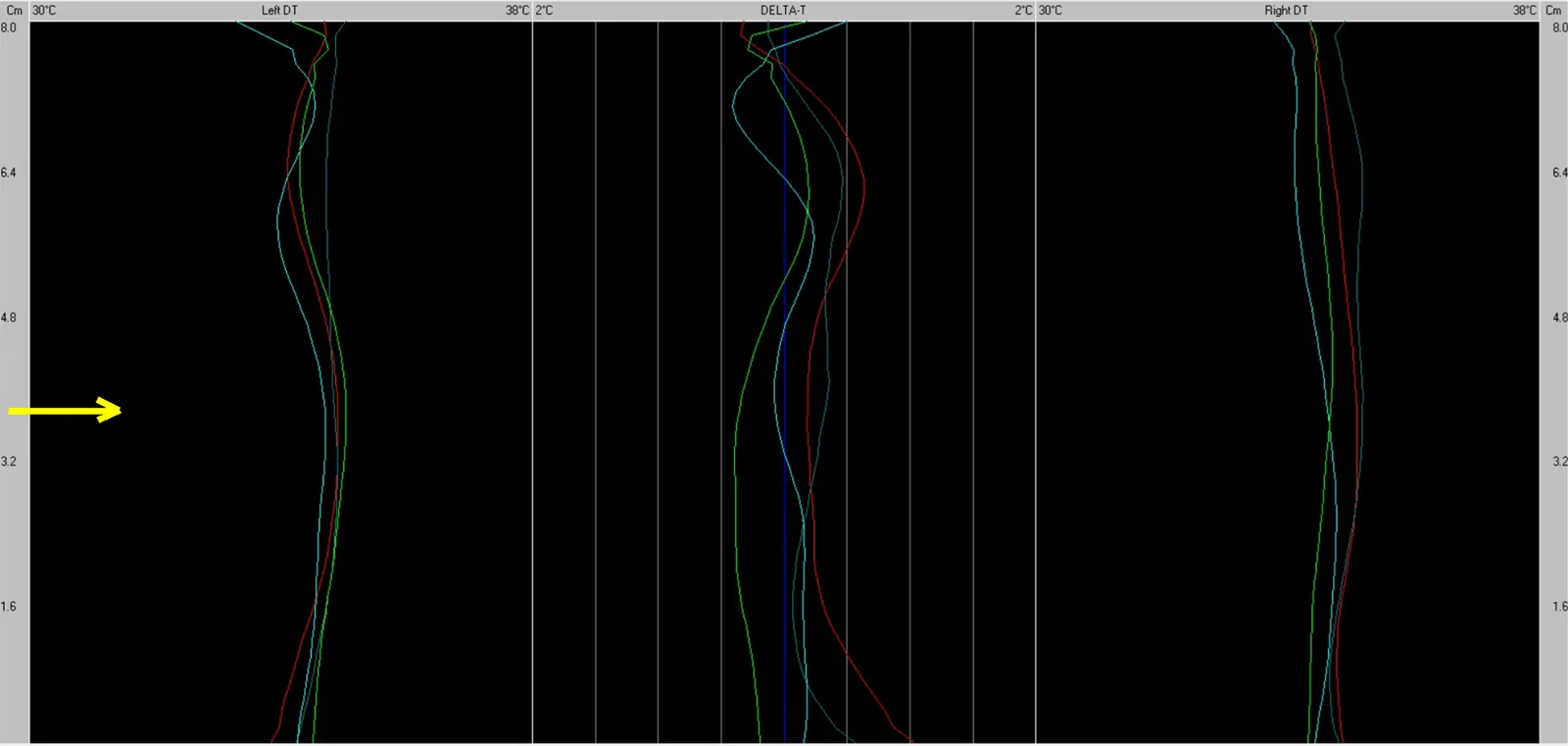
Paraspinal thermographic scans from the initial physical examination (Day 0).

No MRI or CT imaging was obtained. The child was under the ongoing care of her general practitioner and gastroenterologist for the duration of this presentation, and advanced neuroimaging was raised with the attending physician by the family at our suggestion; the physician did not consider it clinically indicated at that time. This is broadly consistent with published guidance indicating that routine neuroimaging is not required in children with recurrent headache and a normal neurological examination [11], although the presence of vomiting in this case is a feature that some guidance flags for closer consideration.

Diagnosis

The child's headaches were characterised by her general practitioner as tension-type headache versus possible migraine: a differential, not a confirmed diagnosis. Her abdominal symptoms were attributed by her gastroenterologist to gastritis; this represented a working diagnosis based on reported symptoms and stool analysis alone, without endoscopic or biopsy confirmation, and should likewise not be read as a confirmed diagnosis. Our own assessment, based on postural, orthopaedic, and radiographic findings, identified findings consistent with upper cervical joint dysfunction. Examination findings of this kind are not pathognomonic for any single condition and were interpreted alongside, rather than in place of, the medical impressions above.

Treatment and outcomes

KCUC correction was initiated using an instrument-assisted (activator) technique, in view of the patient's age. Once this was well tolerated, care transitioned to gentle manual adjustment. Care was initially delivered twice weekly, reducing to once weekly; 12 visits were planned for the initial phase of care, intended to span approximately two months but ultimately delivered over three months due to scheduling. Ten more follow-up/maintenance visits followed before the patient relocated overseas. The total duration under care was approximately 16 months. Recommended visit frequency for the maintenance phase was every two weeks, spacing out as symptoms and physical examination findings remained stable; in practice, gaps of four to six weeks between visits occurred on a number of occasions, including in the period preceding the recurrence of abdominal symptoms at approximately Month 6.

Table 1 summarises the clinical course.

**Table 1:** Timeline of clinical course. KCUC: knee chest upper cervical chiropractic

Day	Event
Day 0	Baseline thermographic scan performed
Day 2	Cervical spine radiographs obtained
Day 0	Initial KCUC visit; instrument-assisted (activator) correction used given patient's age
Day 21	Mild post-adjustment soreness reported; resolved quickly (adverse event)
Day 0–58	Stomach pain reported to improve, per parent
Day 86	Parent reported resolution of arm/hand pain prior to scheduled reassessment
Day 98	12th-visit reassessment: reduced pain and numbness in arm, improved stomach pain, improved posture and head carriage, normalised arm strength and leg length, residual restriction only in left lateral flexion. The family opted to defer a routine follow-up medical appointment scheduled for that date owing to symptomatic stability, while the patient remained under the general care of her medical team.
~Month 6	Recurrence of stomach pain reported by parent, coinciding with a period of inconsistent visit frequency (gaps of 4–6 weeks versus the recommended interval)
~Month 16	Patient relocated overseas; discharged from care after approximately 16 months under supervision, with referral to a local chiropractor

Thermographic scanning was repeated at the 12th visit reassessment (Figure 7). At this visit (Day 98), the patient showed reduced arm pain and numbness, improved abdominal pain, improved posture with reduction in anterior head carriage and head tilt, normalised arm strength on manual muscle testing, and equal leg length; cervical range of motion remained restricted only in left lateral flexion. The family opted to defer a routine follow-up medical appointment scheduled for the same date owing to symptomatic stability, while the patient remained under the general care of her medical team.

**Figure 7:**
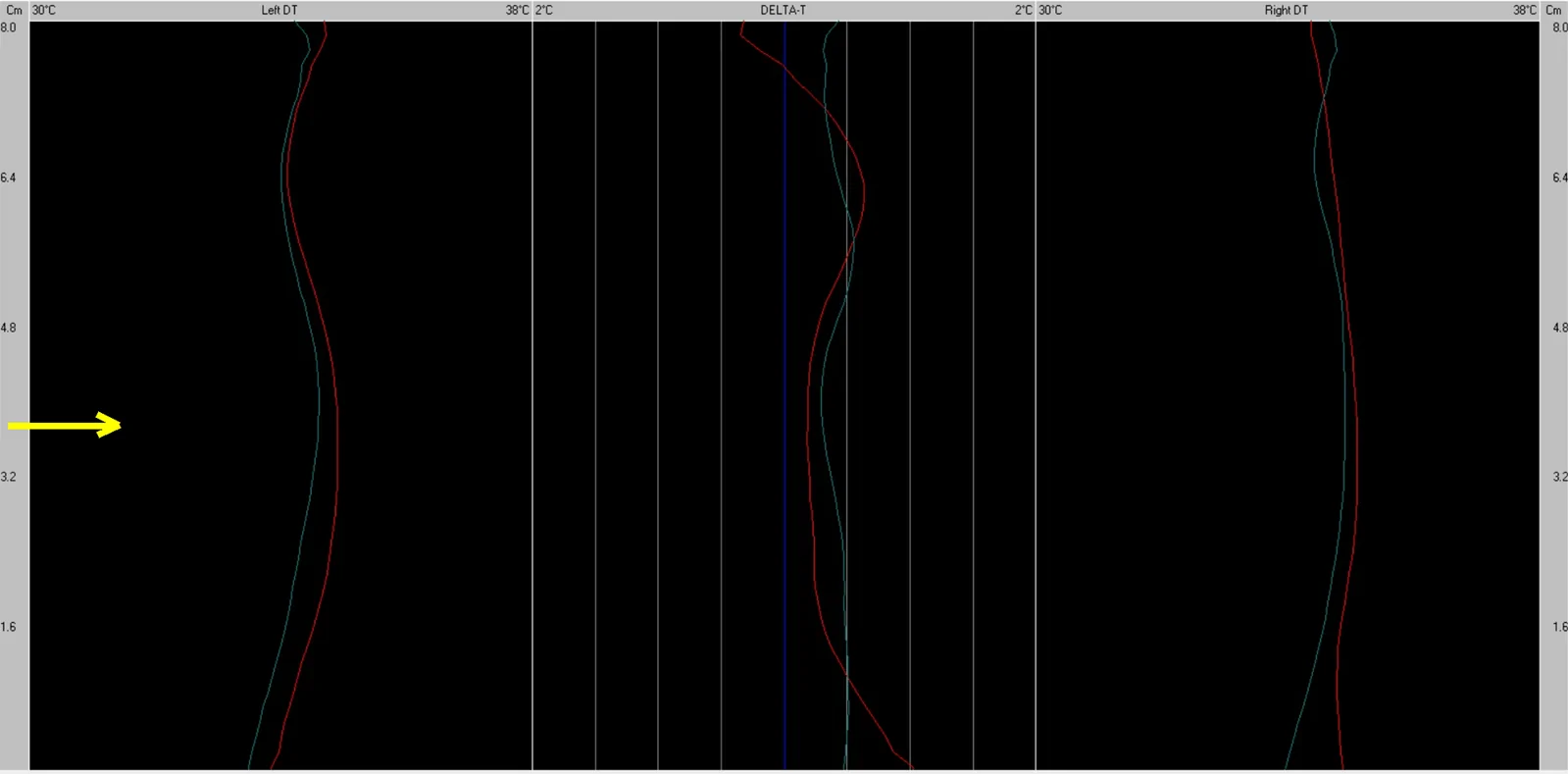
Paraspinal thermographic comparison between initial presentation and the 12th-visit reassessment (Day 98).

Abdominal pain was reported by the mother to have improved over the first two months of care (Day 0-58), before recurring; it improved again by the Day 98 reassessment. Arm/hand pain was reported as resolved by the mother on Day 86, ahead of the scheduled reassessment. This unprompted update, ahead of the scheduled 12th-visit reassessment, was consistent with the objective improvement in arm strength and resolution of arm/hand pain documented at that reassessment. Abdominal pain recurred at approximately Month 6, a period the mother's report and clinic records associate with inconsistent visit adherence; however, we cannot exclude other unrecorded changes in the child's medical management, school situation, or life circumstances over the same period, and this temporal association should not be interpreted as evidence of a therapeutic effect.

Discharge

The patient was discharged from active care when the family relocated overseas. Thermographic scanning was repeated at this point (Figure 8), and she showed temporally associated improvement in her presenting symptoms compared with initial presentation. She had been seen by her general practitioner and gastroenterologist prior to and throughout chiropractic care, and was referred to a local chiropractor to continue monitoring following relocation.

**Figure 8:**
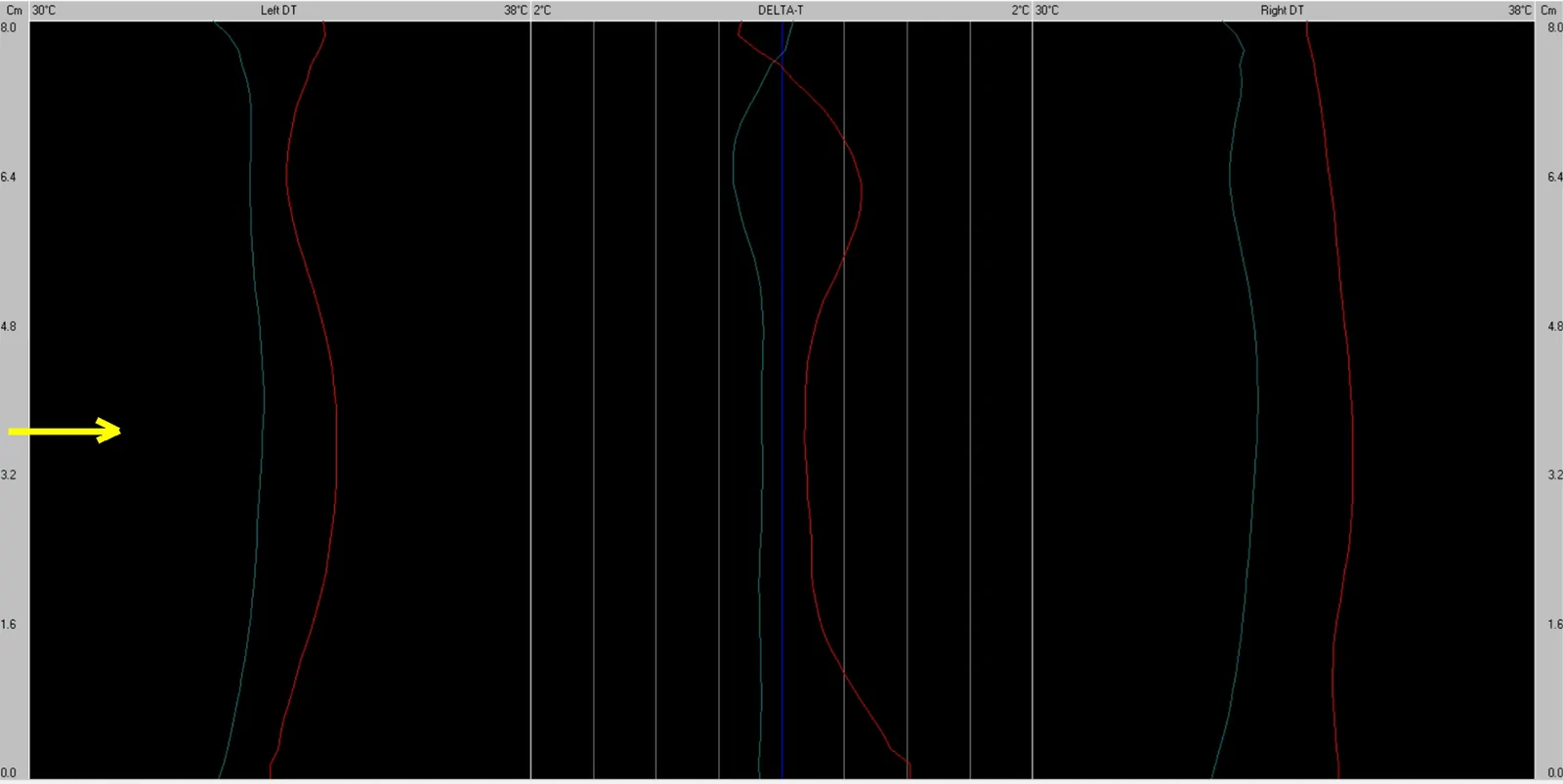
Paraspinal thermographic comparison between initial presentation and the final scan recorded prior to the patient's relocation overseas (Day 0 to month 16)

Adverse events

Mild soreness was reported following adjustment on Day 21, which resolved quickly without intervention. This is consistent with published safety data describing upper cervical chiropractic adverse reactions as generally mild in intensity and short in duration [12]. No other adverse events were reported during the period of care.

## Discussion

This report describes the case of an eight-year-old patient presenting with an unconfirmed migraine-type headache and functional gastrointestinal symptoms, managed with KCUC care alongside ongoing conventional medical supervision. Paediatric headache and recurrent abdominal pain frequently co-occur and are recognised within episodic syndromes associated with migraine [1]; however, published accounts of chiropractic management in this specific paediatric symptom combination are limited to isolated case reports [7], which, to our knowledge, makes this an uncommon presentation in the chiropractic case-report literature. We wish to be explicit about the narrow nature of this contribution: this case describes a child with an undiagnosed symptom complex who received chiropractic care alongside conventional medical management and whose symptoms fluctuated over time. This clinical course is not, in itself, unusual, and it does not provide strong evidence for any specific mechanistic hypothesis; its value lies in documenting a sparsely reported paediatric symptom combination and in generating a hypothesis for future, controlled study, rather than in demonstrating a treatment effect.

The temporal association between periods of consistent chiropractic care and symptomatic improvement, and between reduced visit adherence and symptom recurrence at approximately Month 6, is notable but must be interpreted cautiously. Any proposed mechanism linking upper cervical joint dysfunction to migraine-type headache or gastrointestinal symptoms in this case is hypothetical; no causal relationship can be established from a single case. Several confounding factors could equally explain the pattern observed: the natural fluctuation of paediatric functional abdominal pain and headache, the ongoing medical management provided throughout by the general practitioner and gastroenterologist, the medications taken over this period (paracetamol, ibuprofen, and an anti-emetic), and the non-specific effects of regular clinical contact. We also cannot exclude the possibility that the recurrence of abdominal pain at Month 6 reflected a change in the child's medical management, school situation, or other life circumstances not captured in our records, rather than the reduced visit adherence with which it coincided. Without controlling for these factors, this temporal association cannot be interpreted as evidence of a therapeutic effect.

As a single paediatric case, its findings are not generalisable, and no causal or therapeutic claim can be made. Neither the headache nor the abdominal symptoms in this case were confirmed diagnoses: the headache was described only as a differential of tension-type headache versus possible migraine, and the gastritis diagnosis was a working impression based on reported symptoms and stool analysis, without endoscopic or biopsy confirmation. This case therefore describes the clinical course of a child with an undiagnosed symptom complex, not a child with confirmed diagnoses of migraine and gastritis, and this should be borne in mind when interpreting every subsequent point below.

No MRI or CT was obtained to exclude central pathology; this reflects the attending physician's clinical judgement rather than an assessment made by the treating chiropractor. While general paediatric guidance does not routinely require neuroimaging in children with recurrent headache and a normal neurological examination [11], the combination of headache with vomiting, neck pain, and radiating arm symptoms and numbness in this case are features that could raise concern for increased intracranial pressure or a structural lesion, and their absence remains a significant limitation of the available diagnostic workup. Because central pathology was not excluded, the reported symptomatic improvement could reflect the natural history of an undiagnosed condition rather than any effect of chiropractic care, and this alternative explanation cannot be ruled out on the evidence available.

Outcome assessment in this case also has notable limitations. No standardised, validated paediatric outcome measure was administered, for example, the Paediatric Migraine Disability Assessment (PedMIDAS) for headache-related disability [13] or the Paediatric Quality of Life Inventory (PedsQL) for broader symptom and quality-of-life burden [14], and outcome is instead described narratively based on clinical findings and parental report. Parental report of symptom improvement is subject to recall bias and may be influenced by a desire to report positive outcomes, and no symptom diary or other method independent of the treating practitioner was used to track outcomes in this case; we recommend that future case documentation in this population incorporate such independent tracking where feasible.

The reported improvements in posture, range of motion, and manual muscle testing were assessed by a single, non-blinded treating practitioner and are therefore subject to observer bias. Thermography was used only as an adjunct assessment tool and is not a validated outcome measure for headache, migraine, or gastrointestinal symptoms; we cannot determine from this case whether the thermographic changes observed added meaningful information beyond the clinical examination, or whether they simply tracked it. Likewise, examination findings such as restricted range of motion, leg-length asymmetry, and muscle testing changes are consistent with, but not diagnostic of, upper cervical joint dysfunction; leg-length assessment and manual muscle testing in particular are not validated diagnostic tests for this purpose, their reliability and validity are not well established, and these findings are not pathognomonic for any specific diagnosis. Finally, the patient was also lost to further structured follow-up after relocating overseas, limiting assessment of longer-term outcome.

## Conclusions

This report describes the case of a paediatric patient with an unconfirmed migraine-type headache and functional gastrointestinal symptoms who showed temporally associated improvement during a period of KCUC care delivered alongside ongoing conventional medical supervision. Given the limitations of a single, non-blinded case without a validated outcome measure or advanced imaging, and given the range of plausible confounding explanations, including the natural history of an undiagnosed condition, these observations should be regarded as hypothesis-generating only, and must not be read as evidence of a therapeutic effect. The specific, narrow contribution of this case is to document an uncommon paediatric symptom combination managed alongside conventional care; any hypothesis it generates regarding upper cervical joint dysfunction and paediatric migraine-type headache or gastrointestinal symptoms would require testing in a prospective, controlled study using validated paediatric outcome measures before it could inform clinical practice.
